# Receptor Targeting Using Copolymer-Modified Gold Nanoparticles for pCMV-Luc Gene Delivery to Liver Cancer Cells In Vitro

**DOI:** 10.3390/ijms25095016

**Published:** 2024-05-04

**Authors:** Mkhuseli Zenze, Moganavelli Singh

**Affiliations:** Nano-Gene and Drug Delivery Laboratory, Discipline of Biochemistry, University of KwaZulu-Natal, Private Bag X54001, Durban 4000, South Africa; 213515339@stu.ukzn.ac.za

**Keywords:** gold nanoparticles, liver targeting, lactobionic acid, chitosan, polyethylene glycol, gene expression

## Abstract

The formulation of novel delivery protocols for the targeted delivery of genes into hepatocytes by receptor mediation is important for the treatment of liver-specific disorders, including cancer. Non-viral delivery methods have been extensively studied for gene therapy. Gold nanoparticles (AuNPs) have gained attention in nanomedicine due to their biocompatibility. In this study, AuNPs were synthesized and coated with polymers: chitosan (CS), and polyethylene glycol (PEG). The targeting moiety, lactobionic acid (LA), was added for hepatocyte-specific delivery. Physicochemical characterization revealed that all nano-formulations were spherical and monodispersed, with hydrodynamic sizes between 70 and 250 nm. Nanocomplexes with pCMV-Luc DNA (pDNA) confirmed that the NPs could bind, compact, and protect the pDNA from nuclease degradation. Cytotoxicity studies revealed that the AuNPs were well tolerated (cell viabilities > 70%) in human hepatocellular carcinoma (HepG2), embryonic kidney (HEK293), and colorectal adenocarcinoma (Caco-2) cells, with enhanced transgene activity in all cells. The inclusion of LA in the NP formulation was notable in the HepG2 cells, which overexpress the asialoglycoprotein receptor on their cell surface. A five-fold increase in luciferase gene expression was evident for the LA-targeted AuNPs compared to the non-targeted AuNPs. These AuNPs have shown potential as safe and suitable targeted delivery vehicles for liver-directed gene therapy.

## 1. Introduction

Hepatocellular carcinoma is the sixth most common cancer globally and a significant cause of cancer deaths worldwide, accounting for more than 800,000 incidences and 700,000 deaths each year [1]. Therapeutic gene delivery has been applied to the liver, lungs, brain, and bone marrow with varying degrees of success. Hepatitis B and C (HBV) is a chronic viral disease that affects millions of people worldwide and can lead to cirrhosis and hepatocellular carcinoma (HCC) if untreated. Since over 70% of patients do not benefit from interferon alpha’s standard of care, liver transplantation is a treatment option. Unfortunately, the wait for a donor organ often leads to patient mortality [2], and even if transplantation is successful, the requirement for lifelong immunosuppression raises concerns about vulnerability to further illness [3]. The use of gene-based therapies to treat liver diseases has piqued the interest of researchers, who believe that the best method to treat inborn metabolic errors of the liver is to replace the defective gene with its functional counterpart [4,5].

Nanotechnology has altered many areas of our lives and sparked worldwide interest among academic and industrial researchers. Nanomedicine has introduced nanoparticles (NPs) into the clinical setting and has wholly revolutionized diagnostics and therapeutics. Due to their advantageous physiochemical characteristics, gold nanoparticles (AuNPs) have become one of the most favored and versatile NPs for use in gene delivery. This metallic NP is well known for being simple to synthesize, adaptable in size, easy to functionalize, low in toxicity, having unique optical properties, and being biocompatible [6]. Surface modifications of the AuNPs can improve circulation, lessen cytotoxicity, boost cellular uptake, and enable the attachment of therapeutic nucleic acid molecules [7,8].

Chitosan (CS) is a natural polymer abundantly found in the exoskeleton of crustaceans. It is a polysaccharide derivative of chitin that is organic, nontoxic, biodegradable, and biocompatible [9]. Chitosan is similar in structure to glycosaminoglycans found in the extracellular matrix of cartilage, adding to its benefits in a nano-formulation [10,11]. Due to the interaction between their active amino groups and the negatively charged metal NPs, CS has also developed into a popular polymer coating [12]. The amino groups are protonated in dilute acidic aqueous solutions (pH 6), which increases its solubility [13] and gives CS a high cationic potential and a strong affinity for negatively charged nucleic acids, enabling the formation of ionic complexes [14]. Polyethylene glycol (PEG) is a wildly used polymer in NP formulations for medical applications. It is commonly used due to its ability to improve an NP’s stability and circulation time. Circulation time is enhanced by minimizing interaction between the NP and the blood components and avoiding recognition by the immune system and blood clearance. PEG further improves the NP’s biocompatibility and reduces its cytotoxicity [15].

Tumor cells are known to overexpress molecules, such as receptors, on their cell surfaces compared to normal healthy cells [2]. This can be exploited using active targeting strategies that take advantage of receptor–ligand interactions and utilize receptor-mediated endocytosis for site-specific therapeutic agent delivery [16,17,18]. Lactobionic acid (LA) is a combination of gluconic acid and galactose. LA is recognized for its distinctive characteristics, including its biocompatible and biodegradable ability. Asialoglycoprotein receptors (ASGPR) are known to be overexpressed on the surface of hepatocytes and have a strong preference for galactose residues [19,20]. LA can be employed as a targeted ligand to interact with ASGPR on liver cells since it contains a galactose moiety. The liver-targeting potential of LA makes it a favorable targeting moiety in NP formulations for gene and drug delivery systems [4]. Although similar studies using LA have been conducted previously with selenium NPs [6,21], this current integrated formulation comprising AuNPs, CS, PEG, and LA has not been investigated for gene delivery.

This proof of principle study addresses the need for a suitable gene delivery vehicle using AuNPs, functionalized with CS, PEG, and LA, to efficiently deliver the reporter gene pCMV-Luc DNA (pDNA) to liver cancer cells in vitro.

## 2. Results

### 2.1. Gold Nanoparticle Synthesis, Nanocomplex Formulation, and Characterization

**Figure 1:**
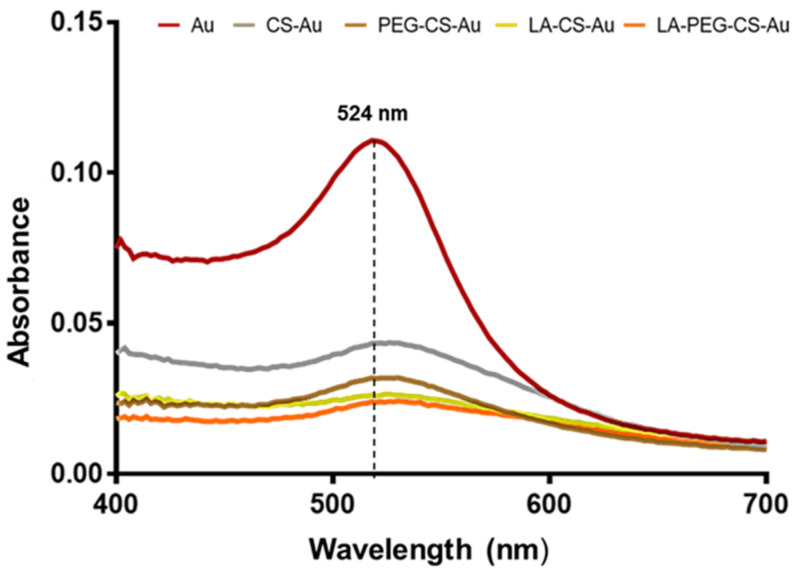
UV-vis spectra of the AuNPs and functionalized AuNPs.

Synthesis of the AuNPs via citrate reduction was successfully achieved, followed by stabilization of the NPs with chitosan (CS) and polyethylene glycol (PEG). The UV–visible (UV-vis) absorption spectra (Figure 1) show the typical absorbance peak for citrate-reduced colloidal AuNPs with a well-defined absorption band and a characteristic λ_max_ at 524 nm. This value agreed with the surface plasmon resonance (SPR) absorption of nanosized gold particles [18]. The adsorption of CS onto the surface of the AuNPs was confirmed by a reduction in the absorbance peak intensity and a redshift in wavelength, which also denoted an increase in particle size [21,22].

**Figure 2:**
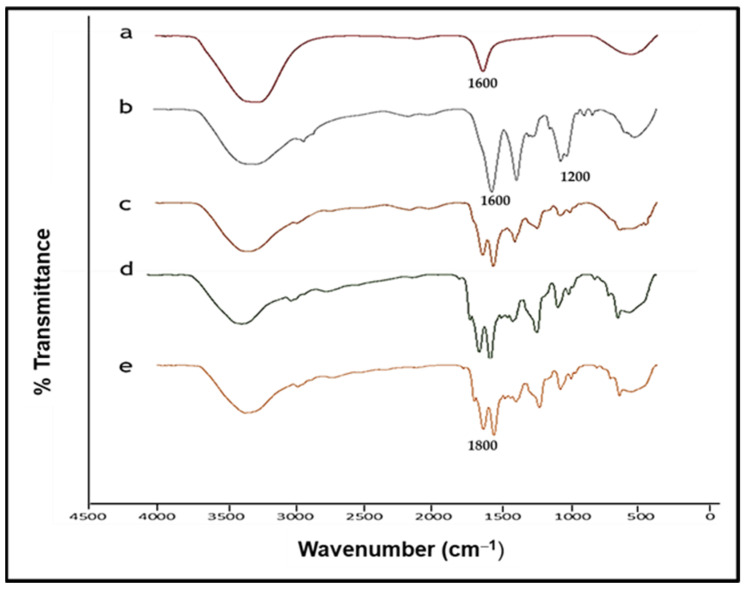
FTIR spectra of (a) AuNP, (b) CS-Au, (c) PEG-CS-Au, (d) LA-CS-Au, and (e) LA-PEG-CS-Au.

FTIR further confirmed the synthesis of the AuNPs and the functionalized AuNPs (FAuNPs) (Figure 2).

The AuNPs exhibited a peak at 1500–1700 cm^−1^, which is in the C=O stretch region. The broad peak below 3500 cm^−1^ suggests the presence of NH_2_ and amide bonds, which are likely related to surface functionalization or ligand attachment on the NPs as noted for the FAuNPs. The CS-AuNPs exhibited a broad peak around 3300–3500 cm^−1^, corresponding to NH_2_ stretching vibrations, which is often broadened due to hydrogen bonding. The peaks around 2800–3000 cm^−1^ are due to C–H stretching vibrations in the CH_2_ and CH_3_ groups of chitosan. The peak around 1650–1655 cm^−1^ is assigned to the amide I band, corresponding to C=O stretching vibrations in the acetylated (amide) groups of chitosan. The peak around 1550–1600 cm^−1^ is assigned to the amide II band, corresponding to N–H bending vibrations coupled with C–N stretching vibrations. The peak around 1030–1150 cm^−1^ is attributed to the stretching vibrations of the glycosidic linkage (C–O–C) in CS. These peaks represent the major functional groups and structural features of CS. The PEG-CS-AuNPs exhibited characteristic signals around 1400 cm^−1^ assigned to C–O, 1106 cm^−1^ attributed to C–O–C, 1755 cm^−1^ ascribed to C=O, and 2996 cm^−1^ due to CH_2_ stretching vibrations. Absorption bands were observed for the LA-PEG-CS-AuNPs between 3200 and 3500 cm^−1^ (O–H and N–H stretching vibrations), 1086 cm^−1^ (C–O stretching vibration), 1633 cm^−1^ (C–O stretching vibration), and 1600 cm^−1^. The AuNPs and all FAuNPs showed a broad peak below 3500 cm^−1^ corresponding to the NH_2_ and amide bonds. Peak assignments are in accordance with those reported in the literature and provide valuable insight into the chemical composition and surface functionalization of the nanoparticles [4,22,23,24].

The size and zeta (ζ) potential of the AuNPs, FAuNPs, and their nanocomplexes with pDNA were assessed using NTA (Table 1). The AuNPs exhibited a hydrodynamic diameter of 77 nm, with an increase in size upon functionalization. All NPs were <175 nm in size. This increase in size correlates with the shift in peaks of the UV spectra, which suggests successful functionalization of the AuNPs. The nanocomplexes with the pDNA exhibited an increase in the hydrodynamic diameter exceeding 200 nm for the PEG-CS-Au nanocomplexes compared to the uncomplexed NPs. The PDI values of the NPs and their nanocomplexes were below 0.4, suggesting a monodisperse population as seen under TEM.

The ζ potential provides insight into the stability of the NPs in a colloidal system. Regardless of the charge, NPs with a ζ potential of ≥20 mV are considered stable [25,26]. The AuNP and FAuNP preparations were all colloidally stable with ζ potentials > 24 mV (Table 1). Functionalization of the AuNPs with CS was further confirmed by a change in the zeta potential from negative for the AuNPs (−33 mV) to positive. CS is rich in positively charged amine moieties and contributed to the higher positive zeta potential measurements for the NPs, further enhancing cellular uptake [27]. The nanocomplexes with pDNA exhibited moderate to high colloidal stability, with the increase in hydrodynamic size and reduction in the positive ζ potential of the FAuNPs being indicative of successful pDNA binding [28].

**Figure 3:**
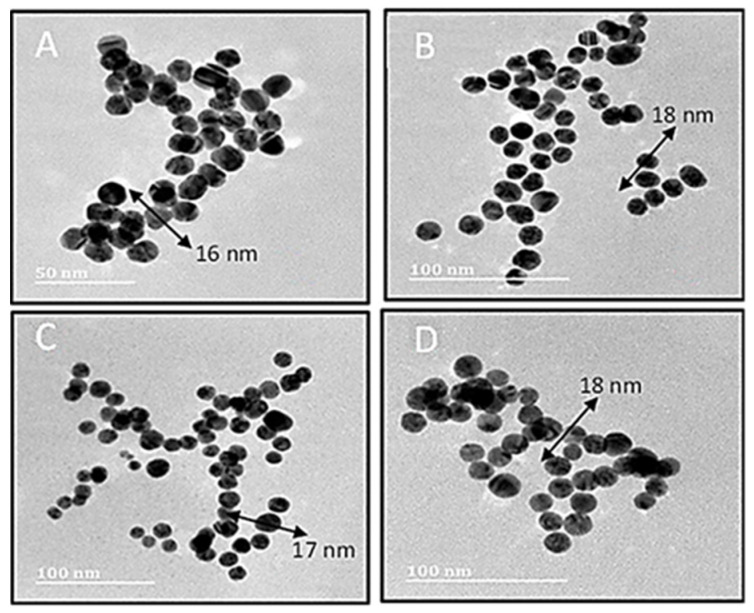
TEM images of nanoparticles: (**A**) CS-Au, (**B**) PEG-CS-Au, (**C**) LA-CS-Au. (**D**) LA-PEG-CS-Au. Scale bar = 50 nm (**A**) and 100 nm (**B**–**D**).

Transmission electron microscopy (TEM) further assessed the size and ultrastructural morphology of the AuNPs and their nanocomplexes (Table 1, Figure 3). TEM images revealed no significant difference in appearance between the AuNPs and the FAuNPs CS-AuNPs. AuNPs appeared to be highly monodisperse, with sizes varying from 13 nm to 18 nm. The nanocomplexes were also found to be similar and monodisperse, with sizes varying from 20 to 25 nm. The slightly larger sizes could be due to the conjugation of the pDNA to the FAuNPs. Subsequent observations showed no structural modifications, such as aggregation or absorption changes for the particles.

### 2.2. Nucleic Acid Binding Studies

**Figure 4:**
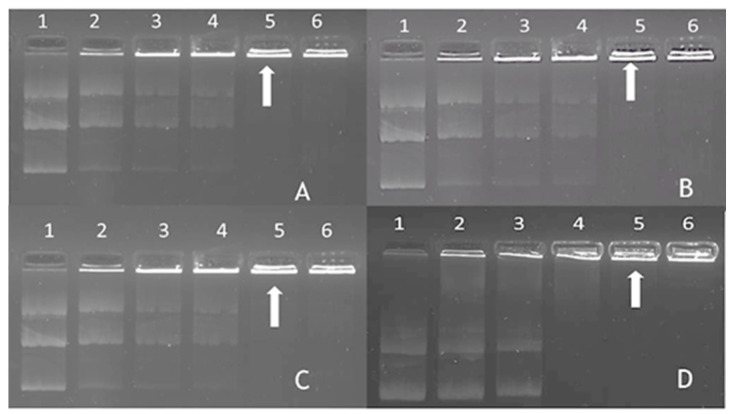
Gel retardation assay of nanocomplexes. pCMV-Luc DNA was kept constant (0.25 µg) in all lanes. (**A**) CS-Au NPs, lanes 1–6 (0, 0.25, 0.4, 0.5, 1.5, and 2.5 µg). (**B**) PEG-CS-Au NPs, lanes 1–6 (0, 0.25, 0.5, 1, 2, and 2.5 µg). (**C**) LA-CS-Au NPs, lanes 1–6 (0, 0.25, 0.5, 1, 1.5, and 2.5 µg). (**D**) LA-PEG-CS-Au NPs, lanes 1–6 (0, 0.25, 0.5, 1, 1.5, and 2 µg). Arrows indicate optimal pDNA binding.

Complex formation between the FAuNPs and the pDNA was analyzed by the commonly used gel retardation assay, which determines the minimum amount of the NP required to bind and neutralize the anionic charges of the pDNA completely. Thus, this produces electroneutral complexes that cannot migrate during electrophoresis and remain in the wells [29,30]. The pDNA was kept constant (0.25 µg) while the FAuNP mass (µg) was varied. Figure 4 shows that the FAuNPs completely bound the pDNA at relatively low ratios. The optimum, sub-optimum, and supra-optimum binding ratios are given in Table 2.

**Figure 5:**
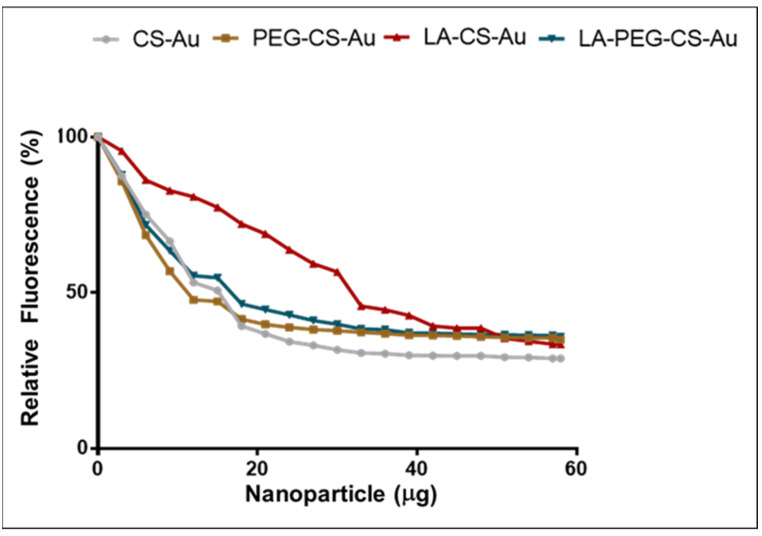
The ethidium bromide intercalation assay. The incubation mixture in HBS (100 μL) contained pCMV-Luc plasmid DNA (2.5 μg) and an increasing amount of nanoparticles.

The ethidium bromide (EB) assay further confirmed the binding but, importantly, indicated the nanocomplexes’ compactness. EB intercalates between the base pairs of dsDNA and produces a strong fluorescence. A decrease in fluorescence following the incremental addition of FAuNPs indicated the displacement of the EB due to the binding of the pDNA by the FAuNPs (Figure 5). A plateau in fluorescence was seen, which showed maximum pDNA condensation at that point.

The CS-AuNPs produced greater fluorescence decay levels than the PEGylated and galactosylated AuNPs. This observation of compaction was confirmed with a maximum percentage fluorescence decay of 79.3% for the CS-AuNPs with 70.6%, 65.4%, and 70.3% for the PEG-CS-AuNPs, LA-CS-AuNPs, and LA-PEG-CS-AuNPs, respectively. Overall, all nanocomplexes showed a steady decline in fluorescence, suggesting that the nanocomplexes were stable and efficiently bound the pDNA.

### 2.3. Nucleic Acid Protection Study

**Figure 6:**
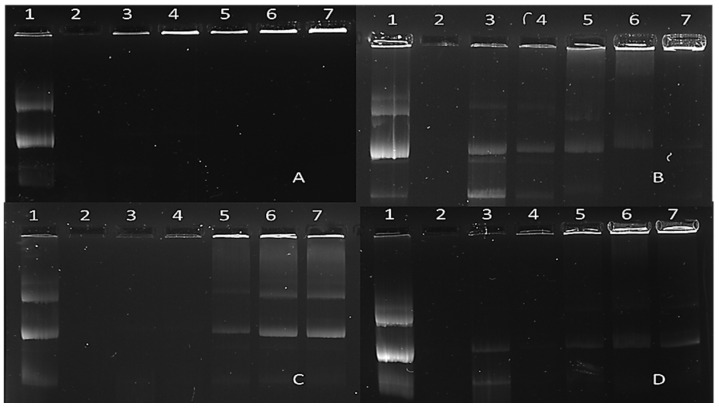
The serum nuclease protection assay of nanocomplexes. Lane 1: positive control (pDNA only); lane 2: negative control (pDNA digested with 10% FBS); lanes 3–4: FAuNP:pDNA binding ratios below the sub-optimum binding ratio; lanes 5–7: FAuNP:pDNA nanocomplexes at the sub-optimum, optimum, and supra-optimum ratios (Table 2). (**A**) CS-AuNP nanocomplexes, (**B**) PEG-CS-AuNP nanocomplexes, (**C**) LA-CS AuNP nanocomplexes, and (**D**) LA-PEG-CS-Au NP nanocomplexes.

To assess the degree of protection offered by the nanocomplexes to degradation by serum nucleases, nanocomplexes were prepared at specific ratios (Table 2), incubated with serum, and subjected to agarose gel electrophoresis. The protection afforded by the FAuNPs to the pDNA is shown in Figure 6.

The positive control (lane 1 of Figure 6A–D) demonstrates the migration of the undigested, intact pDNA. The negative control (lane 2 of Figure 6A–D) displays the fate of naked DNA in the presence of 10 % serum. Lanes 3–7 in Figure 6A–D indicate the migration of the pDNA after incubation of the nanocomplexes with 10% serum. Using the positive and negative controls as a comparison, we can conclude that all FAuNPs at all ratios afforded good protection to the pDNA. Considering this, nanocomplexes were then subjected to in vitro cell-based studies.

### 2.4. Cytotoxicity Assay

**Figure 7:**
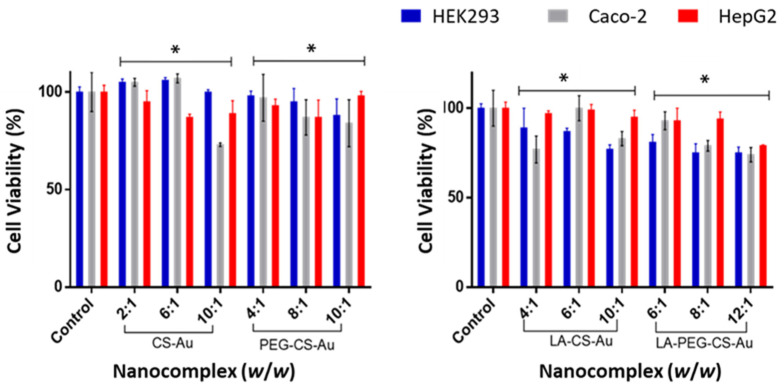
Cell viability of nanocomplexes in the HEK293, Caco-2, and HepG2 cells. Control = untreated cells. Data are represented as mean ± SD (*n* = 3). ^*^ *p* < 0.005 was considered to be statistically significant.

An MTT assay was used to assess the cytotoxicity of the nanocomplexes in three mammalian cell lines: HEK293, Caco-2, and HepG2. A control containing untreated cells representing 100% cell viability was included for each cell line. All nanocomplexes assessed were well tolerated at all ratios, with no specific concentration dependency observed. Maximum growth inhibition of the CS-AuNP and PEG-CS-AuNP nanocomplexes in the receptor-positive HepG2 cells was shown to be 18% and 14%, respectively, at the optimum binding ratio (Figure 7). There was no significant difference for the CS-AuNPs and PEG-CS-AuNPs compared to the control in all cells at all three ratios. However, the CS-Au:pDNA at a ratio of 10:1 exhibited some significant cytotoxicity (above 20%) when compared to the other ratios. Overall, these NPs can be proposed as suitable gene delivery vectors due to their good tolerance in vitro.

The HepG2 cells displayed low toxicity at all binding ratios of the nanocomplexes, with better cell viability recorded for the PEG-CS-Au nanocomplexes at the supra-optimum binding ratio (cell viability of 100%). The receptor-negative HEK293 and Caco-2 cells also showed minimal toxicity, displaying a slight increase of 5% in cell survival at the optimum binding ratio for the CS-Au nanocomplex (Figure 7). This cell proliferation can be attributed to the biocompatibility properties of CS. The targeted LA-CS-AuNP nanocomplex showed a low degree of cytotoxicity (±15%) in the HEK293 cells at the sub-optimum ratio compared to the ±20% displayed by the Caco-2 cells at the same ratio. In the HEK293 and Caco-2 cells, the PEG-CS-Au and LA-PEG-CS-Au nanocomplexes displayed cell inhibition between 15 and 20% at their optimum binding ratios. Overall, we can deduce that all four nanocomplexes exhibited low cytotoxicity in all cells investigated and can be regarded as relatively safe gene delivery vehicles [31]. Although there was negligible cytotoxicity associated with these nanocomplexes, an acridine orange/ethidium bromide (AO/AB) dual staining apoptotic assay was conducted to determine the type of cell death that may have occurred.

### 2.5. Apoptosis Assay

**Figure 8:**
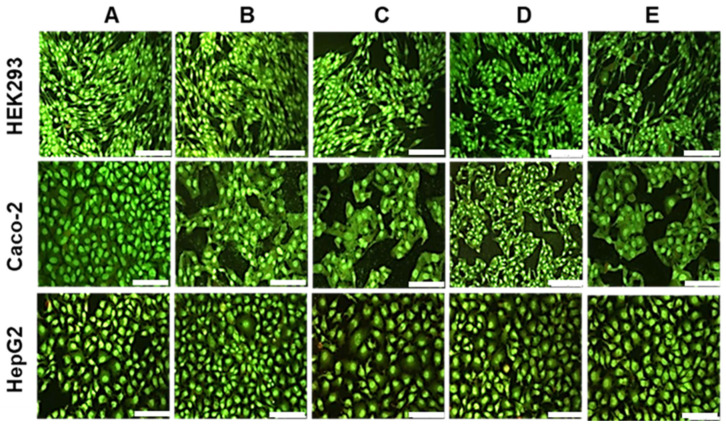
Fluorescent images of the HEK293, Caco-2, and HepG2 cells at 20× magnification. (**A**) control cells (**B**) CS-AuNP/pDNA (**C**) PEG-CS-AuNP/pDNA, (**D**) LA-CS-AuNP/pDNA, and (**E**) LA-PEG-CS-AuNP/pDNA at their optimum binding ratios. Scale bar = 100 μm.

The HEK293, HepG2, and Caco-2 cells treated at the optimum AuNP/pDNA ratio did not exhibit any significant morphological changes or the presence of apoptotic or necrotic bodies (Figure 8). Viable cells appeared green, as seen in all the images obtained. Since no apoptosis was observed, calculating the apoptotic index was unnecessary. These results further confirm the relatively low cytotoxic nature of the formulated nanocomplexes and their potential as safe gene delivery vehicles.

### 2.6. Transgene Expression

**Figure 9:**
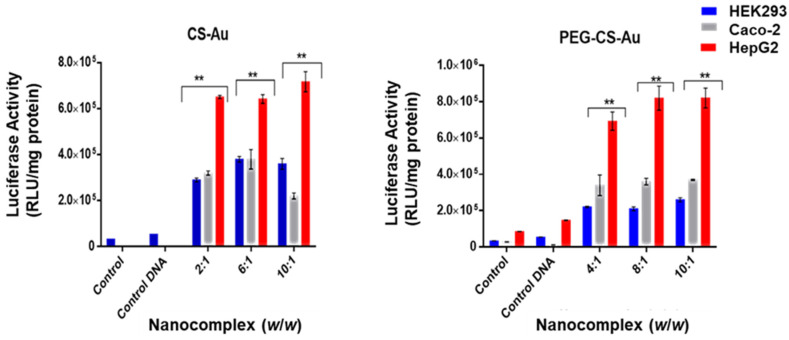
Luciferase activity in HEK293, Caco-2, and HepG2 cells treated with the untargeted nanocomplexes (CS-Au:pDNA and PEG-CS-Au:pDNA). Control = untreated cells, control DNA = naked pDNA. Data are presented as mean ± SD (*n* = 3). ** *p* < 0.001 was considered statistically significant.

**Figure 10:**
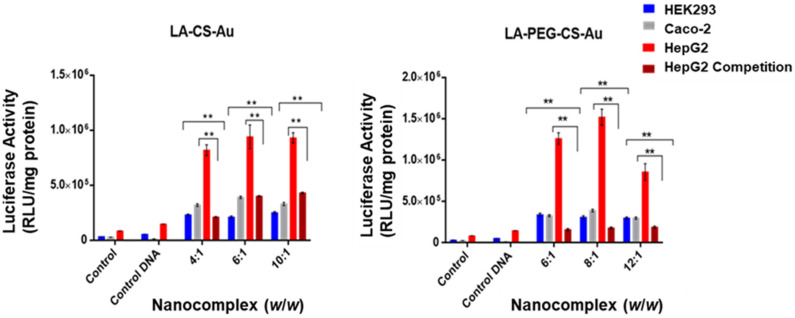
Luciferase activity in the HEK293, Caco-2, and HepG2 cells treated with the targeting nanocomplexes (LA-CS-Au:pDNA and LA-PEG-CS-Au:pDNA). Excess LA was added to the HepG2 cell for the competition assay prior to nanocomplex addition. Control = untreated cells, and control DNA = naked pDNA. Data are presented as mean ± SD (*n* = 3). ** *p* < 0.001 was considered statistically significant.

The luciferase reporter gene (pCMV-Luc DNA) was used to evaluate the transfection activity of all the nanocomplexes. The targeted gene delivery was assessed in the receptor-positive HepG2 cells, with the receptor-negative HEK293 and Caco-2 cells serving as controls to confirm that cellular uptake occurred due to receptor-mediated endocytosis (RME). Two controls were used for this study: cells only and cells treated with naked pDNA. All the nanocomplexes displayed significant transgene expression exceeding that of the controls (Figure 9 and Figure 10). Transfection efficiency was notably higher in the receptor-positive HepG2 cells for all nanocomplexes, with the LA-targeted delivery system (Figure 10) showing significant transfection activity in the HepG2 cells.

CS-Au/pDNA (Figure 9) showed enhanced transfection activity in the HepG2 cells compared to the Caco-2 and HEK293 cells at all three ratios with maximum transfection activity at the supra-optimum ratio (7 × 10^5^ RLU/mg protein). This was a 3- and 5-fold increase in expression compared to the Caco-2 and HEK293 cells (2 × 10^5^ and 3.8 × 10^5^ RLU/mg protein), respectively, at the same binding ratio (10:1 *w*/*w*). For PEG-CS-Au/pDNA nanocomplexes (Figure 9), both the optimum and supra-optimum ratios showed a similar gene expression with a maximum luciferase activity of 9 × 10^5^ RLU/mg protein in the HepG2 cells at both the optimum and supra-optimum binding ratios (8:1 and 10:1 *w*/*w*), respectively). The HEK293 and Caco-2 cells produced lower expression values of 2 × 10^5^ and 4 × 10^5^ RLU/mg protein at the optimum and supra-optimum ratios. PEGylation of the CS-AuNPs produced a 3-fold increase in luciferase expression in the HepG2 cells at a binding ratio of 8:1 *w*/*w* (*p* < 0.01). This correlates with the lower cytotoxicity recorded for the PEGylated nanocomplexes compared to their non-PEGylated counterparts.

Both targeted NP formulations exhibited superior transfection efficiency in the HepG2 cells compared to the Caco-2 and HEK293 cells (Figure 10). The LA-CS-Au/pDNA nanocomplex had the highest luciferase activity of 1 × 10^6^ RLU/mg protein at both the optimum and supra-optimum ratios, with a slight decrease at the sub-optimum ratio in the HepG2 cells. This represented a 4- and 5-fold increase of the expression obtained in the HEK293 and Caco-2 cells at the same ratio. A similar trend was observed in the other cells. The LA-PEG-CS-Au/pDNA nanocomplex (Figure 10) displayed the highest luciferase activity (1.5 × 10^6^ RLU/mg protein) at the optimum ratio in the HepG2 cells, far exceeding that seen in the Caco-2 and HEK293 cells.

**Figure 11:**
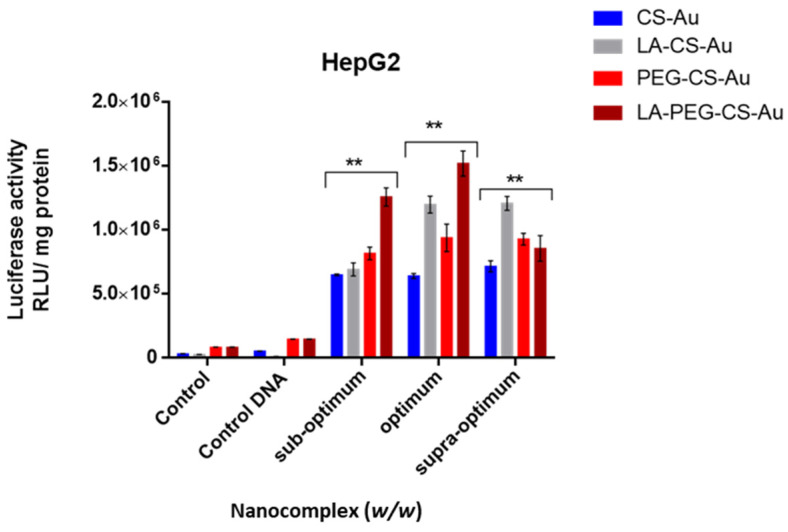
Comparison of the luciferase activity between the targeted and non-targeted nanocomplexes in the HepG2 cells. Control = untreated cells, control DNA = naked pDNA. Data are presented as mean ± SD (*n* = 3). ** *p* < 0.001 was considered statistically significant.

Figure 11 is a consolidated graphical representation showing the difference in the targeted and non-targeted nanocomplexes in the HepG2 cells, confirming that the incorporation of the targeting ligand LA significantly impacted luciferase expression. The high transgene expression elicited by the LA-CS-Au/pDNA and LA-PEG-CS-Au/pDNA nanocomplexes in the HepG2 cells could be attributed to their rapid internalization by RME. To corroborate this claim, a competition assay was conducted to assess if cellular uptake of the targeted nanocomplexes occurred via RME. This was performed by pre-incubating the cells with excess free LA before transfection. The excess LA is bound to the receptors, preventing the LA-CS-Au/pDNA and LA-PEG-CS-Au/pDNA nanocomplexes from entering the cells via receptor mediation. The results showed a significant decline in gene expression (approximately 88%), thus confirming that the uptake of the LA-targeted nanocomplexes was receptor-mediated, as seen in the earlier literature [32]. Furthermore, there was a difference in the transfection activity for the targeted nanocomplexes in the three cell lines. The luciferase activity in the HepG2 cells showed a notable increase in transgene activity compared to that in the receptor-negative cell lines (HEK293 and Caco-2). Hence, clear evidence has been shown that the LA-CS and LA-PEG-CS-AuNPs were successful in cell-specific targeting and are suitable cell-specific carriers of genes.

## 3. Discussion

Synthesis of AuNPs using citrate reduction remains an attractive and reliable method of preparation that uses water and no additional passivating ligand. A recent study revealed that there was very little difference between green and chemical synthesis with regard to gene delivery [33]. CS was used to modify the AuNPs for better stability and for its ability to bind electrostatically to the anionic pDNA via its cationic amine groups. Furthermore, the hydroxyl groups on CS facilitate the conjugation of other molecules [34]. The addition of PEG was to add stability and improve the circulation time of the nanocomplex. At the same time, the conjugation of LA sought to enhance the cell-specific uptake of the nanocomplex via ASGP receptor mediation [4]. UV-vis spectroscopy showed that the AuNPs exhibited absorption peaks within the accepted range for gold. The FAuNPs had a lower peak intensity, suggesting that the respective polymers coated the gold [35]. FTIR analysis is commonly used to detect functional groups and further confirmed the AuNP synthesis and functionalization.

The control of NP size is related to the ratio of the agents used during preparation. The more reducing agents added, the smaller the NP produced. However, the sizes from the NTA were much larger than those from TEM. This could be attributed to the procedures used with TEM, measuring the particles in their dry state, while NTA examines the particles in an aqueous state, which is more in keeping with what one may expect in an in vivo system [34]. Particle size and the shape of the NPs are of major importance, as they influence the internalization of the nanocomplex by the cell. The successful modification of the AuNPs with CS was also evident by the change in size and the positive ζ potential of the AuNPs. All functionalized AuNPs and nanocomplexes had positive ζ potentials that fell within the acceptable range (≥20 mV), except for the LA-PEG-CS-Au and LA-CS-Au, which were slightly lower but >15 mV. This could be due to the masking of charges by the LA on the AuNPs, which leads to fewer cationic groups present. Since ζ potentials are good indicators of colloidal stability [36], these nanocomplexes showed moderate to good stability. The cationic nature of these nanocomplexes can also enhance their uptake by the anionic cell membranes of the cancer cells [37]. The NPs and nanocomplexes had favorable sizes (<200 nm) [38], except for the PEG-CS-Au nanocomplexes, which were slightly over 200 nm. However, this did not affect the uptake of the nanocomplexes, as is evident from the transfection studies. It was reported that for HepG2 cells, the most likely mechanism of cellular uptake of NPs is via clathrin-mediated endocytosis, as the NPs tend to accumulate in the secondary lysosomes [39]. The PDI is generally a good indicator of NP distribution, which can be correlated with that seen under TEM. PDI values <0.1 are assumed to be monodisperse with uniformity in size, while higher PDI values may indicate polydispersity and the potential of the NPs to aggregate [40]. The NPs and nanocomplexes used in this study showed favorable PDI values (<0.1), suggesting a monodisperse NP population.

It has been suggested that electrostatic interactions that occur during nanocomplex formation impact the organization of the solvent present around the complex. This restricts the number of sites exposed for pDNA binding [41]. This shielding of binding sites could be why some nanocomplexes display a higher binding ratio than others. The targeted NPs had similar binding ratios to those of non-targeted NPs, suggesting that LA/PEG were compacted during EDC/NHS coupling and, therefore, could not cause any steric hindrance to the pDNA binding to the CS positive amine groups. This is evident for the CS functionalized AuNPs, which had the same optimal binding ratio as other functionalized AuNPs, such as the LA-CS-AuNPs [42].

EB is a planar aromatic fluorophore whose emission intensity is approximately 10-fold higher when intercalated between nucleic acid base pairs compared to its fluorescence in water or buffer [43]. All the FAuNPs could compact the pDNA by successfully displacing the EB. The LA-PEG-CS-Au and LA-CS-Au showed reduced affinity for the pDNA, with less EB being displaced from the dsDNA. This correlates with their lower zeta potentials. It has been proposed that DNA strand bending can occur during such binding events with AuNPs [44]. High degrees of DNA compaction can be unfavorable for transfection. Although the nanocomplex can safely transport its cargo to the cell, it can become challenging to unpack vectors, which is essential for successful transfection. On the other hand, weak AuNP/pDNA binding can trigger early dissociation, resulting in the degradation of the pDNA. There was no direct correlation between the endpoints of the EB displacement assay and gel retardation assay. The gel retardation assay showed the minimum amount of the NP that was required to bind a fixed amount of pDNA completely. In contrast, the displacement assay was more sensitive and provided information on the strength of the nanocomplex compaction.

When a nanocomplex enters the bloodstream, it is exposed to nucleases in the serum, which can readily digest any unprotected nucleic acid. The ability of an NP to protect DNA is a crucial factor affecting its biological activity [45]. This assay mimics an in vivo system, thus determining the capability of the nanocomplex to protect its cargo when administered systemically. In this study, the NP-bound DNA was mainly in the relaxed closed circular form after incubation with serum, indicating some nicking of the plasmid. It has been reported that complexes with a neutral charge can protect DNA [46], while complexes with an excess positive charge are capable of fully condensing DNA to form a complex that is highly resistant to digestion. The protection of pDNA by the FAuNPs could be attributed to the stability of the complexes and the favorable electrostatic forces between the positively charged NPs and the negatively charged DNA. This leads to highly organized supramolecular structures, where the DNA is condensed or compacted and thus protected mainly against nuclease degradation [47]. Often, the pDNA becomes strongly bound within the nanocomplex, which does not disassociate to release the pDNA after adding sodium dodecyl sulfate, a detergent used to release the pDNA from its nanocarrier. Fluorescent pDNA bands will still be observed in the wells of the gel [29,47]. This was evident for some of the nanocomplexes in this study. These results corroborate those from the EB displacement assay, where high compaction led to good protection of the pDNA.

There are various factors affecting the toxicity of nanocomplexes. These include the nature of the NP, zeta potential, incubation time, type of cell line, and cell density [48]. It has been suggested that cytotoxicity may be due to an increase in cell membrane permeability as well as the creation of transmembrane pores [49]. It has been proposed that there is a distinct synergy between the pDNA and the AuNPs, which affects cell toxicity [45]. The good cell viability for the HepG2 cells agrees with that previously reported, where galactosylated and PEGylated AuNPs displayed low toxicity in HepG2 cells [50]. Low toxicity in the HEK293 cells suggests that these NPs are less likely to harm normal cells and, hence, desirable for nanomedicine. The fluorescent apoptosis assay correlated with the good cell viability observed in the MTT study.

Significant transgene expression was noted for all nanocomplexes in vitro. The HEK293 and Caco-2 cells showed lower expression than the HepG2 cells. The HepG2 cells are known to overexpress the asialoglycoprotein receptor on their cell surface, which has a high affinity for lactobionic acid (LA) which contains galactose residues. Hence, they were used as a suitable model for hepatocyte-targeted delivery. Early studies have shown that HepG2 cells have more than 225,000 asialoglycoprotein receptors [51]. Furthermore, these asialoglycoprotein receptors have shown a specificity for terminal galactose residues as found in many ligands, such as asialofetuin and the lactobionic acid used in this study. Binding to the receptors occurs by the hydrogen bonding of the sugar’s hydroxyl groups with the receptor’s carboxylate and amide side chains [19,20,52]. In order to confirm RME, an excess of LA was added to the HepG2 cells before the targeted nanocomplexes were added. This blocked the asialoglycoprotein receptors, leading to reduced binding of the targeted nanocomplexes and diminished luciferase expression. A similar trend was noted for LA-targeted selenium nanoparticles [4,53]. The results also showed that all non-targeted nanocomplexes displayed better transfection efficiency in the HepG2 cells compared to the HEK293 and Caco-2 cells. This could be attributed to the cellular morphology of hepatocellular cells, which are physiologically designed to absorb foreign compounds much easier and faster compared to other cells. When comparing the Caco-2 and HEK293 cells, at the optimal ratios of all nanocomplexes, the Caco-2 cells exhibited greater transgene expression, which could be due to cancer cells rapidly dividing and hence requiring more nutrients compared to normal cells, as they tend to compete for nutrients with normal cells.

The incorporated CS effectively binds and facilitates pDNA uptake by the cells. Adding PEG to the nanocarriers increases the systemic circulation time, improving gene delivery [52]. It prevents opsonization by proteins that can create a barrier to effective gene delivery [54] while minimizing cellular toxicity, thereby providing the pDNA sufficient time to dissociate from the nanocomplex and be safely delivered into the nucleus [55]. Hence, the PEGylated NPs showed good transfection in this study due to their beneficial properties. Variations in luciferase activity between the NP formulations at the different binding ratios could be attributed to nanocomplex size and stability differences at the various binding ratios [56]. All nanocomplexes produced luciferase expression that was considered statistically significant compared to the control cells at all binding ratios.

Our results indicate that these hepatocyte-targeted NPs are suitable candidates for cell-specific gene delivery and warrant further development.

## 4. Materials and Methods

### 4.1. Materials

Chitosan (25 kDa, 75% deacetylated), lactobionic acid, 1-ethyl-3-(3-dimethylamnopropyl) carbodiimide (EDC) and bicinchoninic acid (BCA), gold (III) chloride trihydrate (Mw: 393.83 g·mol^−1^, HAuCl_4_·3H_2_O), sodium triphosphate (Mw: 367.86 g·mol^−1^), acridine orange hemi (zinc chloride) salt (Mw: 265.36, g·mol^−1^), and dialysis tubing (MWCO = 1000 Daltons) were supplied by Sigma-Aldrich Chemical Co., (St. Louis, MO, USA). Sodium citrate tribasic dehydrate, phosphate-buffered saline tablets [PBS, (140 mM NaCl, 10 mM phosphate buffer, 3 mM KCl)], 3-[(4,5-dimethylthiazol-2-yl)-2,5-diphenyl tetrazolium bromide] (MTT), ethidium bromide, glacial acetic acid, and dimethyl sulfoxide (DMSO), were obtained from Merck (Darmstadt, Germany). Mammalian cells, embryonic kidney (HEK293), colorectal adenocarcinoma (Caco-2), and hepatocellular carcinoma (HepG2) were obtained from the American Type Culture Collection (Pty) Ltd., Manassas, VA, USA. Sterile fetal bovine serum (FBS) was supplied by Hyclone GE Healthcare (Utah, USA). Eagle’s Minimum Essential Medium (EMEM) with L-glutamine (4.5 g·mL^−1^), penicillin/streptomycin/amphotericin B (100×) antibiotic mixture (Amphotericin B (25 mg/mL), penicillin (10,000 Units/mL), streptomycin sulfate (100 mg/mL)], and trypsin versene mixture were supplied by Lonza Bio Whittaker (Verviers, Belgium). Plasmid Factory (Bielefeld, Germany) provided the reporter gene pCMV-Luc DNA (6.2 Kbp). All sterile tissue culture plasticware was obtained from Corning Inc. (New York, NY, USA). All other reagents were of analytical grade, and ultrapure (18 MOhm) water (Milli-Q Academic, Millipore, France) was used throughout.

### 4.2. Preparation of Colloidal Gold Nanoparticles (AuNPs)

AuNPs were chemically synthesized using an adaptation of the Turkevich method [57], involving the chemical reduction of gold (III) chloride trihydrate (HAuCl_4_) with sodium citrate (NaC_6_H_5_O_7_). Briefly, 25 mL of 18 MOhm water was heated under constant stirring to a temperature between 85 and 95 °C. Approximately 375 μL of a 3 × 10^−2^ M of the HAuCl_4_ solution was added, followed by 1 mL of 1% sodium citrate added directly into the vortex of the solution. A color change from pale yellow to dark blue was seen within 5 min, and, finally, a deep wine-red color was produced after 15 min of constant heating and stirring. The solution was stirred for a further 5 min, cooled to room temperature, and stored in a dark bottle. The final concentration of the colloidal AuNPs was 0.45 × 10^−3^ M.

### 4.3. Functionalization of AuNPs with Chitosan (CS) and Polyethylene Glycol (PEG)

The AuNPs were functionalized with a 1 mg/mL solution of CS (in 1% acetic acid). Briefly, 1 mL of the above AuNP suspension was added dropwise to 1 mL of the CS solution under constant stirring for 2 h. The resulting CS-AuNP suspension was then stirred for 24 h at room temperature, followed by dialysis (12,000 MWCO) against ultrapure water for 2 h. The sample was stored at room temperature for further use.

PEGylation of the AuNPs was adapted from that reported earlier [58]. The CS-AuNPs were PEGylated using a 3% mass percentage of polyethylene glycol 2000 (PEG2000). PEG was gradually added to the CS-AuNP suspension under constant stirring, and the resulting PEG-CS-AuNP suspension was stored at 4 °C for further use.

### 4.4. Synthesis of Lactobionic Acid-Chitosan (LA-CS) and PEGylation

The LA-CS conjugate was prepared by crosslinking the primary amines to the carboxylic acid groups. LA (0.5 g) and EDC (0.15 g) were dissolved in DMSO, followed by 25 mL Milli-Q water. After that, 1 mL of 1 mg/mL of CS was added to 1 mL of the LA-EDC solution and stirred for 24 h to ensure maximum LA conjugation and removal of DMSO. Thereafter, 1 mL of colloidal AuNPs was added to the LA-CS solution with stirring for 24 h. The product was dialyzed as in Section 4.3 and stored at 4 °C.

LA-CS-AuNPs’ PEGylation was achieved by first conjugating LA-CS with 3% PEG and EDC/NHS with continuous stirring for 24 h. The AuNPs were added as for the synthesis of the LA-CS-AuNP above. The final PEG-LA-CS-AuNP suspension was stored at 4 °C for further use.

**Figure 12:**
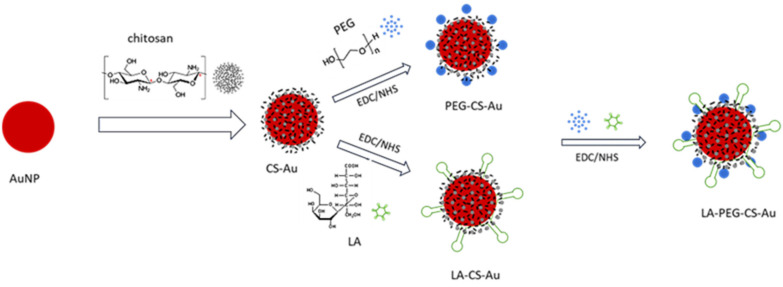
Schematic illustration depicting the synthesis of the functionalized AuNPS.

Figure 12 provides an illustration of the functionalization of the AuNPs with CS, PEG, and LA.

### 4.5. Nanocomplex Formation

NP suspensions were sonicated for 1 min before use. The pCMV-Luc DNA reporter gene (0.25 μg) was added to varied amounts of the different FAuNP formulations to produce a range of FAuNP:pDNA (*w*/*w*) mass ratios. The mixtures were gently vortexed and incubated for 60 min at room temperature for nanocomplex formation.

### 4.6. Nanoparticle and Nanocomplex Characterization

UV-vis spectroscopy was conducted on all synthesized AuNPs, FAuNPs, and nanocomplexes to confirm their absorption spectra. These data were correlated with those in the literature. Samples were assessed using a Biomate 3 spectrophotometer (Thermo Fischer Scientific Inc., Waltham, MA, USA) at a 400–700 nm wavelength range.

TEM was used to obtain the ultrastructural morphology of all samples. Approximately 10 μL of each sample was placed onto a 400-mesh carbon-coated copper grid (Ted Pella Inc. Redding, USA) and air-dried at room temperature for 1 h. Samples were then viewed at 60,000× magnification using a JEOL-JEM T1010 (Jeol, Tokyo, Japan) electron microscope without warming above −150 °C at an acceleration voltage of 100 kV. Images were captured and analyzed using the TEM Soft Imaging Systems (SIS) version 3.1 Megaview III fitted with a side-mounted 3-megapixel digital camera.

The hydrodynamic size distribution, zeta potential, and polydispersity of all NPs and nanocomplexes were analyzed using NTA (Nanosight NS-500, Malvern Instruments, Malvern, UK) at 25 °C. Approximately 1 ml of 1:500 dilutions (in 18 MOhm water) of each sample were prepared and assessed.

FTIR was employed to confirm the presence of essential groups and bonds in the NPs by visualization of the presence of specific peaks. The spectrum was analyzed in a Perkin Elmer Spectrum 100 spectrophotometer from 4000–400 cm^−1^. The IR spectra were obtained using Spectrum Analysis Software version 10.

### 4.7. Nucleic Acid Binding Assays

#### 4.7.1. Gel Retardation Assays

The optimum binding ratios (*w*/*w*) for the FAuNPs were assessed using a gel retardation assay [27]. Nanocomplexes were prepared as in Section 4.5. and Table 3. Gel loading buffer (40% sucrose, 0.5% bromophenol Blue, 2 µL) was added to the nanocomplexes, which were then subjected to agarose gel electrophoresis for 60 min at 50 volts using a 1% agarose gel in a Bio-Rad electrophoresis apparatus containing 1× electrophoresis buffer (36 mM Tris-HCl, 30 mM sodium phosphate, 10 mM EDTA pH 7.5) and 3 µL of ethidium bromide (10 mg/mL). The resultant gel was viewed under UV transillumination at a 300 millisecond exposure time in a Vacutec Syngene G:Box BioImaging system (Syngene, Cambridge, UK).

#### 4.7.2. Ethidium Bromide (EB) Intercalation Assay

An EB intercalation assay was used to evaluate the degree of the FAuNP:pDNA interaction and the FAuNPs’ ability to compact pDNA [27]. Approximately 2 µL EB (100 µg/mL) was dispensed into a 96-well FluorTrac flat-bottomed black plate (Greiner Bio-One, Frickenhausen, Germany) containing 100 µL of HBS. This established a 0% fluorescence baseline measured using a Glomax^®^ Multidetection system (Promega Biosystems, Sunnyvale, CA, USA) at excitation and emission wavelengths of 520 nm and 600 nm, respectively. The pDNA (2.5 µg) was then added to the HBS:EB mixture and this reading was taken as 100% fluorescence. The FAuNPs (1 µL aliquots) were added stepwise with brief mixing, and individual fluorescence readings were recorded until a plateau in readings was noted. The relative fluorescence (Fr) was calculated using Equation 1 [59].

% F_R_ = ((F_i_ − F_0_)/(F_max_ − F_0_)) × 100 (1)

#### 4.7.3. Nuclease Protection Assay

A nuclease protection assay was conducted to assess the ability of the FAuNPs to protect the pDNA from nuclease degradation. The nanocomplexes were prepared at their suboptimum, optimum, and supra-optimum binding ratios, as determined in Section 4.7.1. A positive control of pDNA in the absence of the NPs and DNase 1, and a negative control of pDNA in the presence of DNase 1 but in the absence of the NPs were included. Following nanocomplex formation, DNase 1 (1 μg/μL) was added to a final concentration of 0.1 µg/µL, and the mixtures were incubated for 4 hours at 37 °C. Thereafter, EDTA and SDS were added to the reaction mixtures to a final concentration of 10 mM and 0.5%, respectively. This was followed by a further incubation at 55 °C for 20 min and agarose gel electrophoresis as in Section 4.7.1.

### 4.8. Cell Culture

All tissue culture-based studies were conducted in a class II biosafety laminar flow hood under sterile conditions. Cells were propagated and maintained in 25 cm^2^ tissue culture flasks containing 5 mL of complete medium (EMEM containing 10% (*v*/*v*) FBS) and antibiotics (100 U·mL^−1^ penicillin, 100 μg/mL streptomycin) at 37 °C in a HEPA class 100 Steri-Cult CO_2_ incubator (Thermo-Fisher Corporation, Waltham, MA, USA). Cells were monitored daily under a Nikon TMS inverted microscope (Nikon Corp, Tokyo, Japan). Cells were subcultured into multiwell plates for assays or cryopreserved.

### 4.9. MTT Cytotoxicity Assay

Confluent cells were seeded into 96-well plates at a density of 2.5 × 10^4^ cells per well and incubated at 37 °C for 24 h to allow for the attachment of cells. The medium was then replaced with 100 μL of fresh medium, followed by adding the respective nanocomplexes (Table 2). Positive controls, containing cells only, were included and recorded as 100 % survival. Assays were conducted in triplicate. Cells were incubated for 48 h at 37 °C in 5% CO_2_. The medium was then removed and replaced with 100 μL fresh medium containing 10 μL of MTT (5 mg/mL in PBS). Cells were incubated for 4 h at 37 °C. The MTT medium was removed, and 100 μL of DMSO was added to each well to solubilize the insoluble formazan crystals. Absorbance was read in a Mindray MR-96A microplate reader (Vacutec, Hamburg, Germany) at 570 nm, using DMSO as a blank. The cell viability (%) was calculated using Equation (2).

% F_R_ = ((F_i_ − F_0_)/(F_max_ − F_0_)) × 100 (2)

### 4.10. Apoptosis Assay

This assay is a rapid fluorescent technique for detecting apoptosis in vitro and uses the dual dye system of acridine orange (AO) and EB [60]. The dyes were each prepared to a concentration of 100 mg/mL in PBS and mixed at a ratio of 1:1 (*v*/*v*). Cells were seeded into 24-well plates at a density of 1.2 × 10^5^ cells per well and incubated at 37 °C for 24 h. The medium was then replenished with 400 μL of fresh medium, and the nanocomplexes at their optimum binding ratios (Table 2) were added. Positive controls of untreated cells were used. The assay was performed in triplicate. Cells were incubated for 48 h at 37 °C in 5% CO_2_, washed with cold PBS (100 μL), followed by adding 15 μL of the dual dye to each well. Cells were viewed under an Olympus fluorescence microscope (200× magnification), fitted with a CC1_2_ fluorescence camera (Olympus Co., Tokyo, Japan).

### 4.11. Luciferase Assay

The cells were seeded in 96-well plates at a density of 2.5 × 10^4^ cells per well and incubated at 37 °C overnight, followed by replacement of the medium after that. For this study, two controls were used: untreated cells and cells treated with naked pDNA. The cells were treated and incubated with the nanocomplexes as in Section 4.9. After the 48 h incubation, the cells were washed twice with PBS, followed by adding 80 µL of cell lysis reagent (1×) to the cells. The cells were then gently shaken using a Stuart Scientific Platform Rocker for 15 min at 30 rev/min. The lysed cell suspensions were pelleted by centrifugation at 12,000× *g* for 20 s in an Eppendorf 541D centrifuge (Merck, Hamburg, Germany). Approximately 100 µL of the luciferase reagent was then added to 20 µL of the cell-free extract, mixed, and luminescence (10 s) was assessed using a GloMax^®^-Multi Detection System (Promega BioSystems, Sunnyvale, CA, USA). The protein content of the cell-free extracts was determined using the standard bicinchoninic acid (BCA) assay. This was used to normalize the luciferase activity, and the results were represented as relative light units (RLU) per mg protein.

### 4.12. Statistical Analysis

Experiments were conducted in triplicate, and all data are presented as the mean ± standard deviation (±SD, *n* = 3). Data were analyzed using two-way ANOVA (Tukey test) in GraphPad Prism Version 9.2 (GraphPad Software Inc. Boston, MA, USA). Analysis was conducted for the interaction of row factor and column factor. Statistical analyses were carried out among mean values using Dunnett’s post hoc test. Comparisons were performed between the experimental data and their respective controls. The statistical significance of the tests was set at ** *p* < 0.001 and * *p* < 0.005.

## 5. Conclusions

This study exploited the favorable characteristics and parameters of AuNPs to produce a safe and efficient proof-of-principle nanosystem for the delivery of the pCMV-Luc-DNA. The FAuNPs and their nanocomplexes displayed good stability, low cellular toxicity, and superior transgene activity in hepatocellular carcinoma cells. The addition of LA to the formulation further enhanced their transfection efficacy in HepG2 cells. Adding PEG further stabilized the NPs, resulting in favorable transgene expression in HepG2 cells. Overall, these FAuNPs have shown the potential to serve as non-viral gene delivery systems for hepatocellular carcinoma treatment and can help to address the challenges faced in liver-targeted therapeutic delivery. Further in-depth studies are warranted to investigate the synergistic roles of LA, PEG, CS, and AuNP in the nanosystem and to assess the system using a therapeutic gene in an animal model. Furthermore, observations of their inherent low cytotoxicity bode well for their use as liver-targeted anticancer drug delivery vehicles as well. This could play an important role in preventing the adverse side effects seen in traditional chemotherapy.

## Figures and Tables

**Table 1:** Hydrodynamic size (HD), zeta (ζ) potential, and polydispersity indices (PDIs) of synthesized AuNPs, FAuNPs, and their respective nanocomplexes with pDNA.

Nanoparticles				Nanocomplexes		
NP	NTA Size (nm)	ζ Potential (mV)	PDI	NTA Size (nm)	ζ Potential (mV)	PDI
Au	77 ± 2	−33 ± 0.5	0.00074	-	-	-
CS-Au	145 ± 20	36 ± 0.4	0.078	155 ± 27	21.8 ± 0.4	0.030
PEG-CS-Au	170 ± 21	34 ± 3.0	0.032	213 ± 41	21.9 ± 0.2	0.044
LA-CS-Au	98 ± 5	31 ± 1.0	0.0026	111 ± 22	15.9 ± 0.5	0.017
LA-PEG-CS-Au	103 ± 15	25 ± 0.3	0.023	144 ± 8	19.2 ± 0.2	0.041

**Table 2:** FAuNP:pDNA (*w*/*w*) ratios obtained from the gel retardation assay.

Nanocomplexes	Sub-Optimum	Optimum	Supra-Optimum
CS-AuNP: DNA	2:1	6:1	10:1
PEG-CS-AuNP:DNA	4:1	8:1	10:1
LA-CS-AuNP:DNA	4:1	6:1	10:1
LA-PEG-CS-AuNP:DNA	4:1	6:1	8:1

**Table 3:** Varied amounts (µg) of the FAuNPs used in the gel retardation assay. pDNA was kept constant at 0.25 µg.

CS-Au (µg)	PEG-CS-Au (µg)	LA-CS-Au (µg)	LA-PEG-CS-Au (µg)
0.25	0.25	0.25	0.25
0.4	0.5	0.5	0.5
0.5	1	1	1
1.5	2	1.5	1.5
2.5	2.5	2.5	2

## Data Availability

All data presented in the study are included in the article. Further inquiries can be directed to the corresponding author.
